# Serial changes of renal function after directly acting antivirals treatment for chronic hepatitis C: A 1-year follow-up study after treatment

**DOI:** 10.1371/journal.pone.0231102

**Published:** 2020-04-14

**Authors:** Shao-Ming Chiu, Ming-Chao Tsai, Chun-Yen Lin, Chien-Hung Chen, Sheng-Nan Lu, Chao-Hung Hung, I-Shyan Sheen, Rong-Nan Chien, Chih-Lang Lin, Tsung-Hui Hu, Yu-Fan Cheng, Chao-Long Chen

**Affiliations:** 1 Division of Hepato-Gastroenterology, Kaohsiung Chang Gung Memorial Hospital and Chang Gung University College of Medicine, Kaohsiung, Taiwan; 2 Graduate Institute of Clinical Medical Sciences, Chang Gung University College of Medicine, Taoyuan, Taiwan; 3 Division of Hepato-Gastroenterology, Department of Internal Medicine, Linkou Chang Gung Memorial Hospital and Chang Gung University College of Medicine, Taoyuan, Taiwan; 4 Division of Hepato-Gastroenterology, Department of Internal Medicine, Chiayi Chang Gung Memorial Hospital, Chiayi, Taiwan; 5 Division of Hepato-Gastroenterology, Department of Internal Medicine, Keelung Chang Gung Memorial Hospital, Keelung, Taiwan; 6 Department of Radiology, Kaohsiung Chang Gung Memorial Hospital, Kaohsiung, Taiwan; 7 Department of Surgery, Kaohsiung Chang Gung Memorial Hospital, Kaohsiung, Taiwan; Nihon University School of Medicine, JAPAN

## Abstract

**Background:**

Our preliminary data showed a slight decrease of estimated glomerular filtration rate (eGFR) after direct-acting antivirals (DAAs) treatment in chronic hepatitis C (CHC). However, long-term outcome of renal evolution after DAAs has not been well documented.

**Aim:**

To assess the renal function under DAAs treatment in CHC patients of an Asian population at 6 months and 1 year after complete treatment.

**Methods:**

A cohort of 1536 CHC patients who received therapies with DAAs were analyzed. Serial eGFR levels at 24 weeks after treatment (SVR_24_) and 48 weeks after treatment (SVR_48_) were evaluated. We compared eGFR at baseline, SVR_12_, SVR_24_ and SVR_48_, and defined renal function deterioration as decrease of eGFR >25% from baseline to SVR_24_ and SVR_48_.

**Results:**

Overall, there was decline of eGFR from SVR_12_ to SVR_48_ in all patients (84.30 ± 27.00 -> 73.20 ± 28.67 mL/min/1.73m^2^, *p*<0.001). This trend of decline was similar in all groups. Multivariate analysis for deterioration in renal function from baseline to SVR_24_ showed liver transplantation, hypertension and baseline eGFR < 60 mL/min/1.73m^2^ were independent risk factors. Multivariate analysis for persistent deterioration in renal function from baseline to SVR_48_ showed liver transplantation, baseline eGFR < 60 mL/min/1.73m^2^ and DCV/ASV use were independent predictive factors.

**Conclusions:**

There is a trend of decline in eGFR at 1-year after DAAs treatment regardless of baseline renal function or DAAs. Liver transplantation and baseline eGFR < 60 mL/min/1.73m^2^ were independent predictive factors of persistent deterioration in renal function from baseline to SVR_48_. Close monitoring renal function in these patients was suggested.

## Introduction

Chronic hepatitis C virus (HCV) infection is a worldwide problem, that affects about 71 million people globally. Approximately 399000 people die from hepatitis C each year, mostly from cirrhosis or hepatocellular carcinoma (HCC). [1] Therefore, effective anti-viral therapy is important to these patients. Over these years, choices of treatment had evolved year by year. New direct-acting anti-viral (DAA) regimens offered effective, well-tolerated treatment to patients with chronic HCV infection who were considered difficult to treat in the past. [2]

In addition to liver disease, recent studies demonstrated that chronic HCV infection also affect kinds of organs other than the liver. The relationship between HCV and chronic kidney disease (CKD) is also under investigation. [3] Among the currently approved DAAs, sofosbuvir is the only one that has significant renal elimination. The other currently approved DAAs–daclatasvir, ombitasvir, paritaprevir/ritonavir, simeprevir, ledipasvir, dasabuvir, grazoprevir and elbasvir—are not eliminated by kidneys. Therefore, in severe CKD or hemodialysis (HD) patient, dose adjustment might not be needed. [4] There are less data on the safety of DAAs in patients with moderate to severe renal function impairment, as the majority of the clinical studies excluded patients with advanced kidney disease. Our previous study has shown a slight decline of estimated glomerular filtration rate (eGFR) at end of treatment (EOT) of DAAs, followed by a slight rise 12 weeks after treatment (SVR_12_) [5]. However, long-term effect of renal toxicity exerted by DAAs has not been well defined. The aim of this study is to assess the renal function under DAAs treatment in patients of an Asian population with chronic HCV infection at half year (SVR_24_) and 1 year (SVR_48_) after treatment.

## Materials and methods

### Study design and patient population

This retrospective cohort study was inducted by collecting data from four institutions of Chang Gung Medical Hospitals in Taiwan (the Keelung, the Linkou, the Chiayi and the Kaohsiung Chang Gung Memorial Hospital). This study protocol had previously been approved by the ethical committees of Chang Gung memorial Hospital (IRB number 201900673B0). The requirement for informed consent was waived by the IRB. The data were analyzed anonymously. All CHC patients who were naïve with treatment of DAAs between Jan. 2015 and Dec. 2017 were identified and reviewed to confirm its feasibility. These regimens included sofosbuvir (SOF) or combination with ledipasvir (LDV), paritaprevir/ritonavir, ombitasvir, dasabuvir (ProD), daclatasvir plus asunaprevir (DCV/ASV), and grazoprevir plus elbasvir (GRZ/EBR). The use of DAAs was determined by the individual physicians’ decision according to a nationwide government-funded program Taiwan. The inclusion criteria were treatment with at least 12 or 24 weeks DAAs. Those who met the following criteria were excluded: history of end stage renal disease under hemodialysis therapy, organ transplantation (liver transplantation was not excluded), human immunodeficiency virus (HIV) infection, chronic hepatitis B infection, hepatitis D infection and loss of follow-up.

### Assessment of renal function

Assessment of renal function was based on eGFR using the isotope dilution mass spectrometry (IDMS) traceable Modification of Diet in Renal Disease (MDRD) at 24 weeks after treatment (SVR_24_) and 48 weeks after treatment (SVR_48_) from Jan. 2015 to Dec. 2017. The IDMS-MDRD equation is: eGFR = 175 x (creatinine)^− 1.154^ x (age)^− 0.203^ x (0.742 if female) x (1.212 if patient is black). Serum creatinine data was recorded from outpatient department.

As we had already known, serum creatinine concentration changes with physiological variability in true GFR. [6] Tiny fluctuations in eGFR are common even in a healthy adult, and it may not necessarily indicate disease progression. To overcome this problem, the Kidney Disease Improving Global Outcomes organization has recommended that renal function progression be defined as change in eGFR category combined with a minimal percentage of decrease in eGFR (25% or greater). [7] In the present study, we defined renal function deterioration as decrease of eGFR >25% from baseline to SVR_24_, and persistent renal function deterioration as decrease of eGFR >25% from baseline to SVR_48_. The category of eGFR was classified according to eGFR values applying cut-off values by the study [8]. We also defined percentage of eGFR change as SVR_48_ minus baseline then divided by baseline, in order to present the amplitude of change in eGFR.

### Data analysis

Data are presented as means ± SD, proportions, or median (range). The differences in continuous and categorical variables across the four groups were assessed using ANOVA and Chi-square, as appropriate. The change in eGFR among SVR_12_, SVR_24_ and SVR_48_ was analyzed using the repeated measures ANOVA to compare the changes trend. Multivariate logistic regression models were used to identify factors associated with renal function deterioration. All statistical analyses were performed using SPSS 22.0. All *p* values of < 0.05 were accepted as statistically significant.

## Results

### Baseline characteristics of the study population

A total of 1536 CHC DAA-naive patients treated in four institutions of Chang Gung Medical Hospital in Taiwan (the Keelung, the Linkou, the Chiayi and the Kaohsiung Chang Gung Memorial Hospital) were included for this study. Of them, 835 persons were in the ProD group, 265 in DCV/ASV group, 218 in SOF-based group, and 218 in GRZ/EBR group. Overall, 45%, 59%, and 14% of subjects were male, liver cirrhotic, and had HCC, respectively. The majority of patients were infected with genotype 1b HCV (88%). The patients of SOF-based group had higher level of AST, ALT, total bilirubin and HCV RNA than others. On the other hand, the patients of GRZ/EBR group had higher level of baseline creatinine than others. Detailed baseline characteristics of the four study groups are presented in Table 1.

**Table 1:** Baseline characteristics of the study population.

	Total (n = 1536)	ProD (n = 835)	DCV/ASV (n = 265)	SOF-based (n = 218)	GZP/EBV (n = 218)	*p*-value
Age (years)	65.2 ± 10.2	64.5 ± 10.2	66.9 ± 9.8	61.9 ± 9.5	63.0 ± 10.1	<0.001
Male gender, n (%)	695 (45.2%)	394 (47.2%)	106 (40.0%)	91 (41.7%)	104 (47.7%)	0.120
Hemoglobin (g/dl)	13.4 ± 1.9	13.7 ± 1.7	13.1 ± 1.9	12.6 ± 2.3	13.2 ± 1.6	<0.001
AST (U/L)	83.0 ± 59.2	81.2 ± 51.7	82.0 ± 63.9	93.5 ± 65.4	80.4 ± 71.7	0.046
ALT (U/L)	99.4 ± 107.6	92.5 ± 71.4	86.8 ± 79.3	127.7 ± 223.9	78.3 ± 59.1	<0.001
Total bilirubin (mg/dL)	1.1 ± 0.9	1.0 ± 0.6	1.0 ± 0.5	1.5 ± 1.8	1.0 ± 0.7	<0.001
HCV-RNA (10^5^ IU/mL)	2.6 ± 5.5	2.5 ± 3.0	2.2 ± 4.5	4.4 ± 10.4	2.0 ± 3.0	<0.001
Diabetes mellitus, n (%)	509 (33.1%)	285 (34.1%)	81 (30.6%)	67 (30.7%)	76 (34.9%)	0.567
HCV genotype, n (%)						<0.001
1a	73 (4.8%)	56 (6.7%)	0	11 (5.0%)	6 (2.8%)	
1b	1347 (87.7%)	779 (93.9%)	263 (99.2%)	95 (43.6%)	210 (96.3%)	
2	107 (7.0%)	0	0	107 (49.1%)	0	
4	1 (0.1%)	0	0	0	2 (0.9%)	
6	2 (0.1%)	0	0	3 (1.4%)	0	
mixed	3 (0.2%)	0	2 (0.8%)	2 (1.0%)	0	
Liver cirrhosis, n (%)	889 (58.5%)	494 (59.2%)	181 (68.3%)	97 (44.5%)	127 (58.3%)	<0.001
HCC, n (%)	217 (14.1%)	105 (12.6%)	42 (15.8%)	31 (14.2%)	39 (17.9%)	0.83
Liver transplantation, n (%)	65 (4.2%)	7 (0.8%)	0	58 (26.6%)	0	<0.001
Creatinine (mg/dl)	0.91 ± 0.77	0.91 ± 0.75	0.91 ± 0.63	0.89 ± 0.92	0.98 ± 0.80	0.626
eGFR (mL/min/1.73m^2^)	84.8 ± 25.3	85.7± 24.7	82.3 ± 24.5	87.6 ± 25.2	81.8 ± 28.0	0.026
eGFR category, n (%)						0.016
G1: >90 mL/min/1.73m^2^	637 (41.5%)	347 (41.6%)	105 (39.6%)	108 (49.5%)	77 (35.3%)	
G2: 60–89 mL/min/1.73m^2^	661 (43.0%)	377 (45.1%)	107 (40.4%)	84 (38.5%)	93 (42.7%)	
G3: 30–59 mL/min/1.73m^2^	206 (13.4%)	95 (11.4%)	48 (18.1%)	23 (10.6%)	40 (18.3%)	
G4: 15–29 mL/min/1.73m^2^	18 (1.2%)	8 (1.0%)	3 (1.1%)	2 (0.9%)	5 (2.3%)	
G5: <15 mL/min/1.73m^2^	14 (0.9%)	8 (1.0%)	2 (0.8%)	1 (0.5%)	3 (1.4%)	

Data are expressed as mean ± standard deviation or number (percentage).

Abbreviation: ProD, paritaprevir/ritonavir/ombitasvir/dasabuvir; DCV/ASV, daclatasvir/asunaprevir; SOF, sofosbuvir; GRZ/EBR, grazoprevir/elbasvir; AST, aspartate transaminase; ALT, alanine aminotransferase; HCC, hepatocellular carcinoma; eGFR, estimated glomerular filtration rate

### Comparison of eGFR among DAAs at the time of EOT, SVR_12_, SVR_24_ and SVR_48_

**Fig 1:**
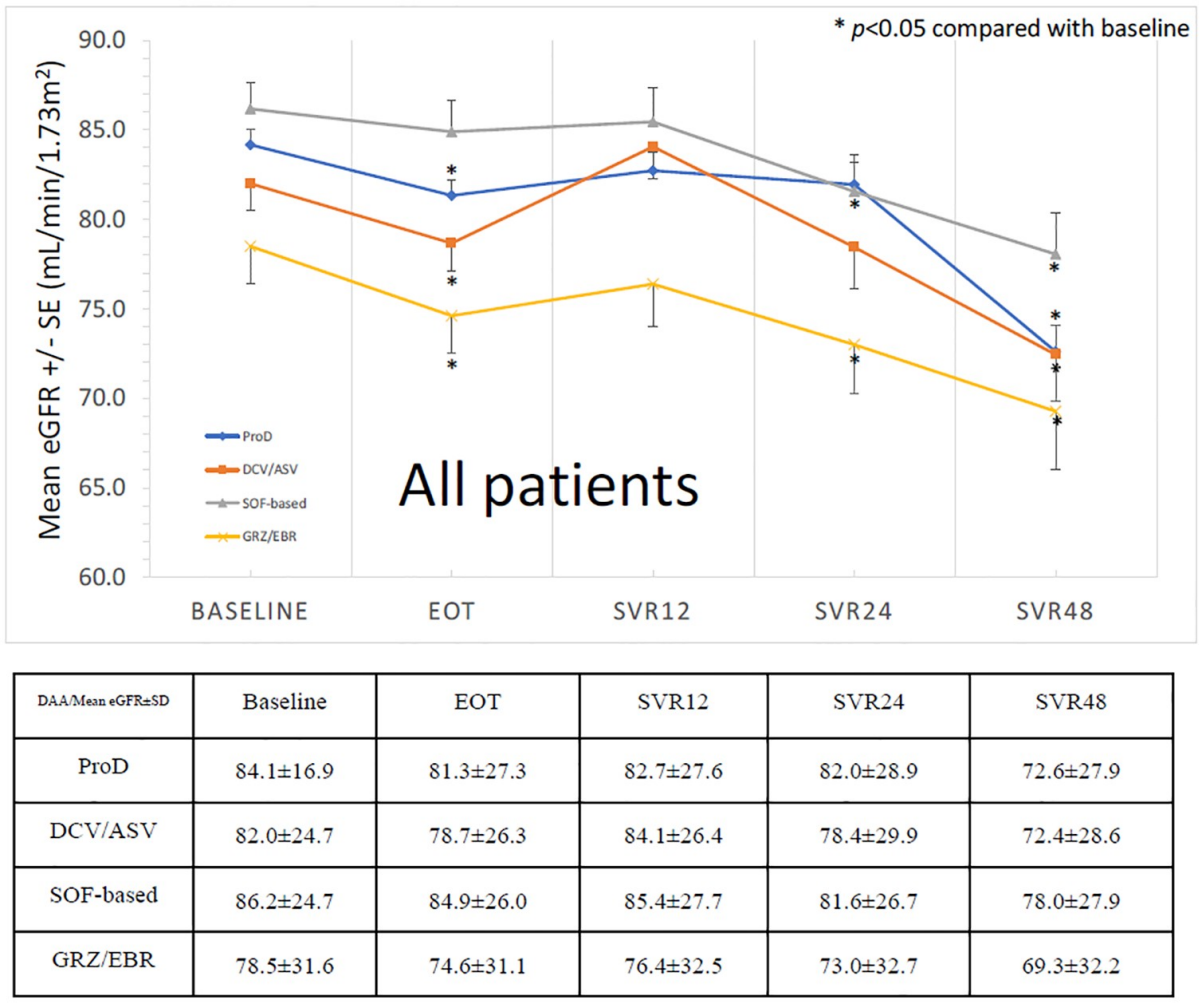
The trend of eGFR from baseline to SVR_48_ (all patients).

**Fig 2:**
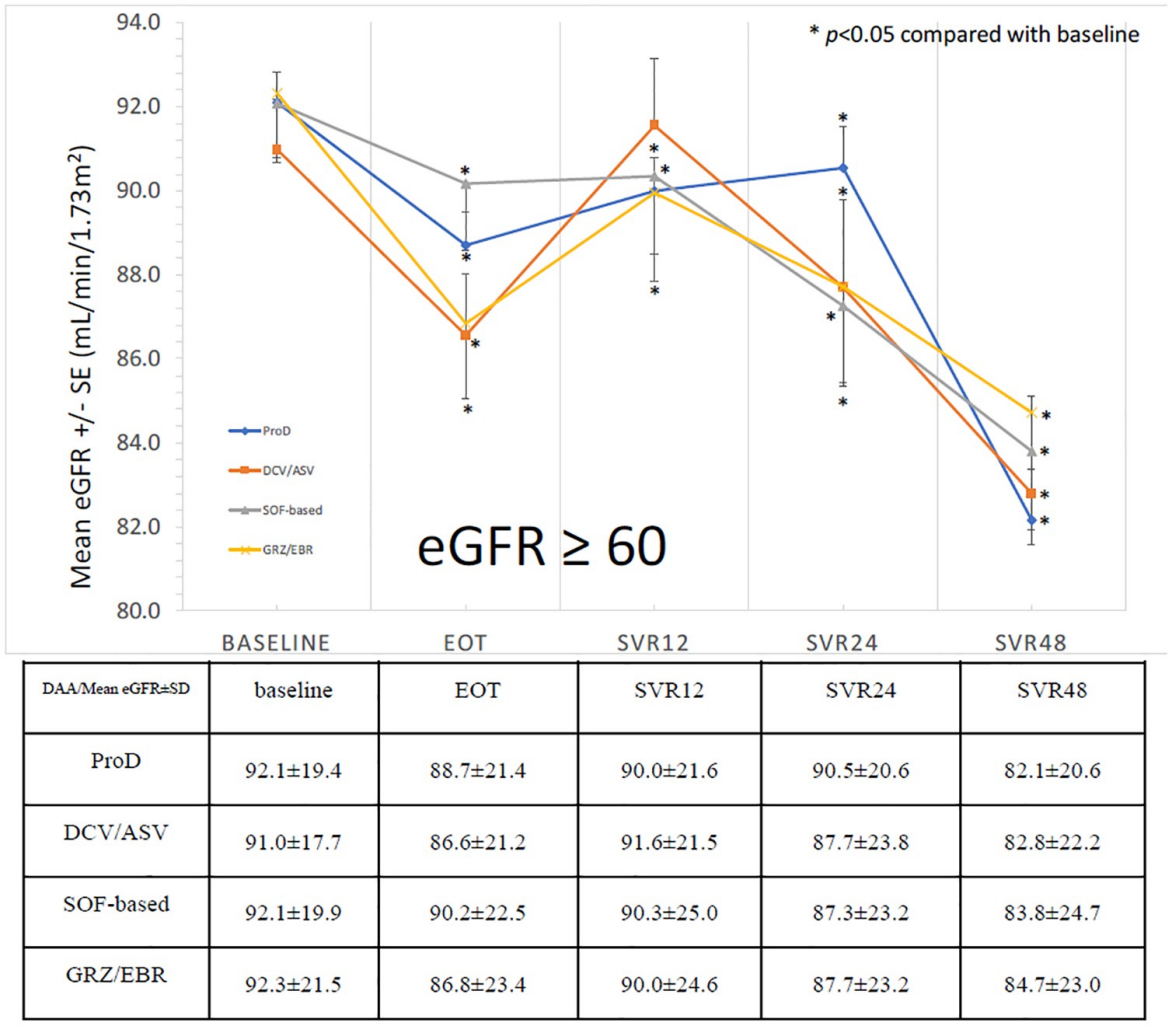
The trend of eGFR from baseline to SVR_48_ (baseline eGFR ≥ 60 mL/min/1.73m^2^).

**Fig 3:**
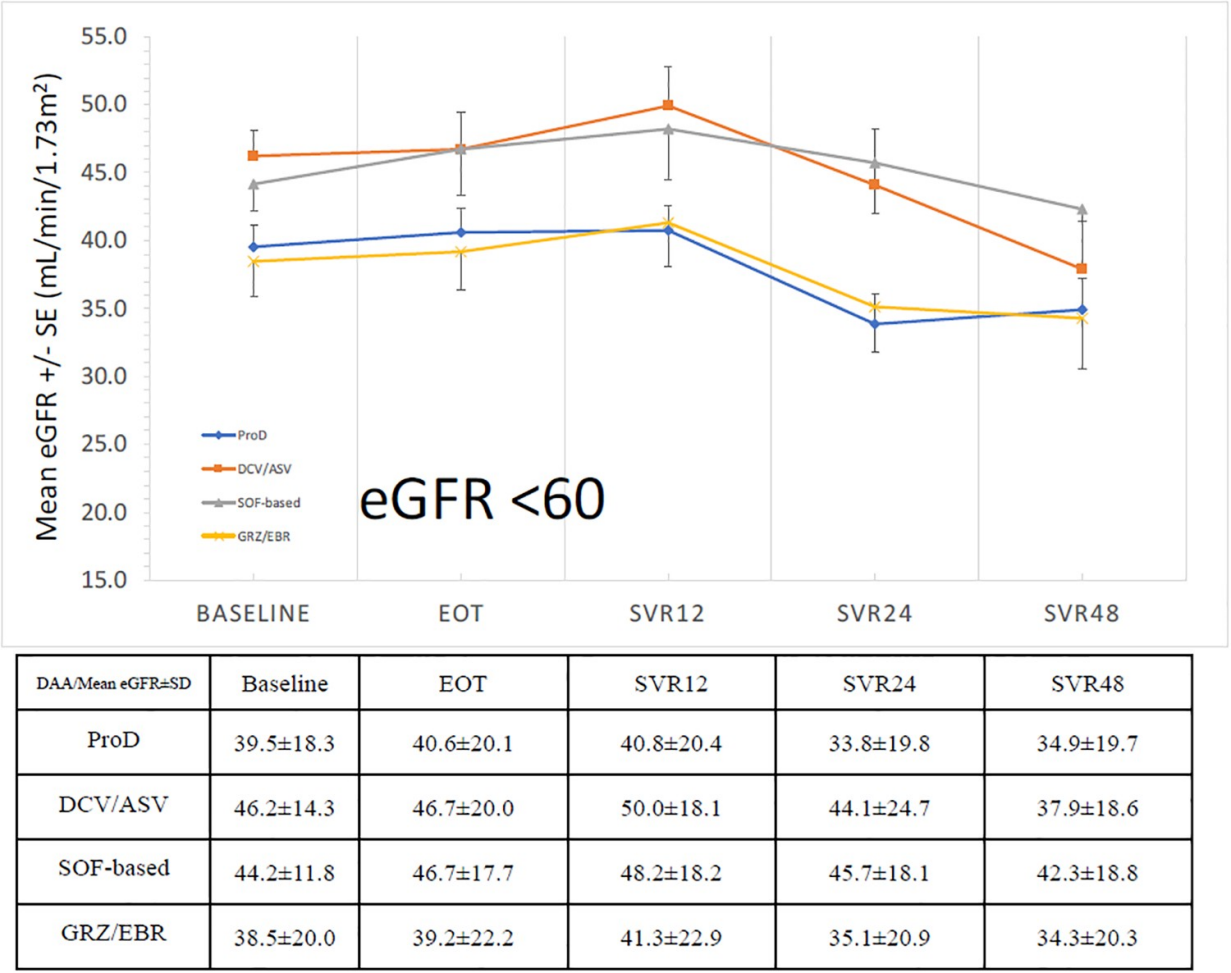
The trend of eGFR from baseline to SVR_48_ (baseline eGFR < 60 mL/min/1.73m^2^).

The evolution of means levels of eGFR from baseline to SVR_48_ was shown as Fig 1. We can see the trend of decrease of eGFR during treatment, then increase of eGFR from EOT to SVR_12_ as described in previous study [5]. Consequently, there is a progressive decline of eGFR again from SVR_12_ to SVR_24_ (84.30 ± 27.00 -> 79.98 ± 29.40 mL/min/1.73m^2^), SVR_24_ to SVR_48_ (79.98 ± 29.40 -> 73.20 ± 28.67 mL/min/1.73m^2^) and SVR_12_ to SVR_48_ (84.30 ± 27.00 -> 73.20 ± 28.67 mL/min/1.73m^2^). This trend of decline was similar in all treatment regimens. In the subgroup analysis of patients with baseline eGFR ≥60 mL/min/1.73m^2^, the trend of eGFR change from baseline to SVR_48_ was the same (91.95 ± 19.48 -> 82.91 ± 22.04 mL/min/1.73m^2^)(Fig 2). In patients with baseline eGFR <60 mL/min/1.73m^2^, the trend of decline was as also similar, but without significant difference from baseline to SVR_48_ (41.17 ± 17.46 -> 36.31 ± 19.50 mL/min/1.73m^2^)(Fig 3).

### The association of percentage of eGFR change (SVR_48_ minus baseline/baseline) with baseline eGFR

**Fig 4:**
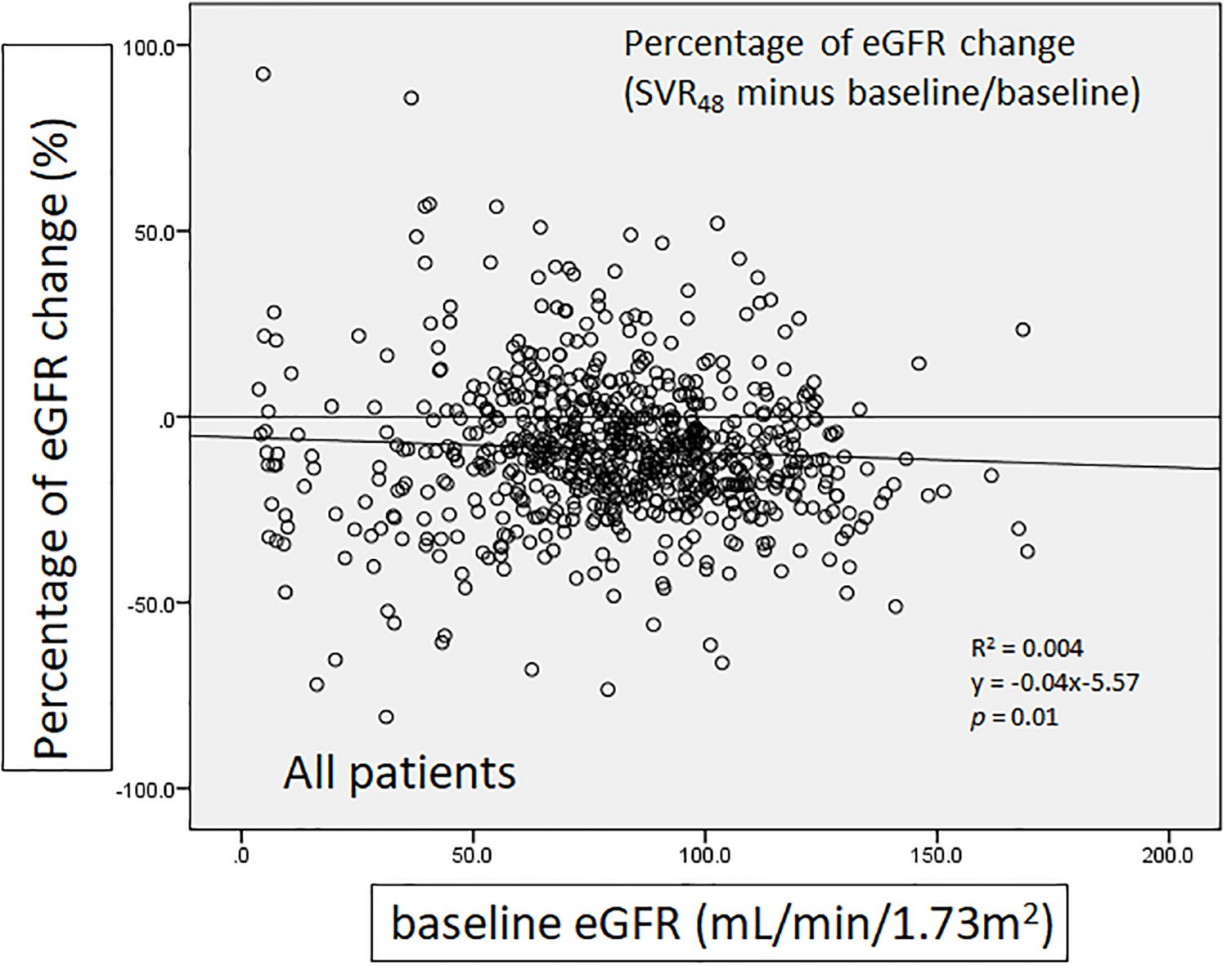
The correlation of percentage of eGFR change with baseline eGFR (all patients).

**Fig 5:**
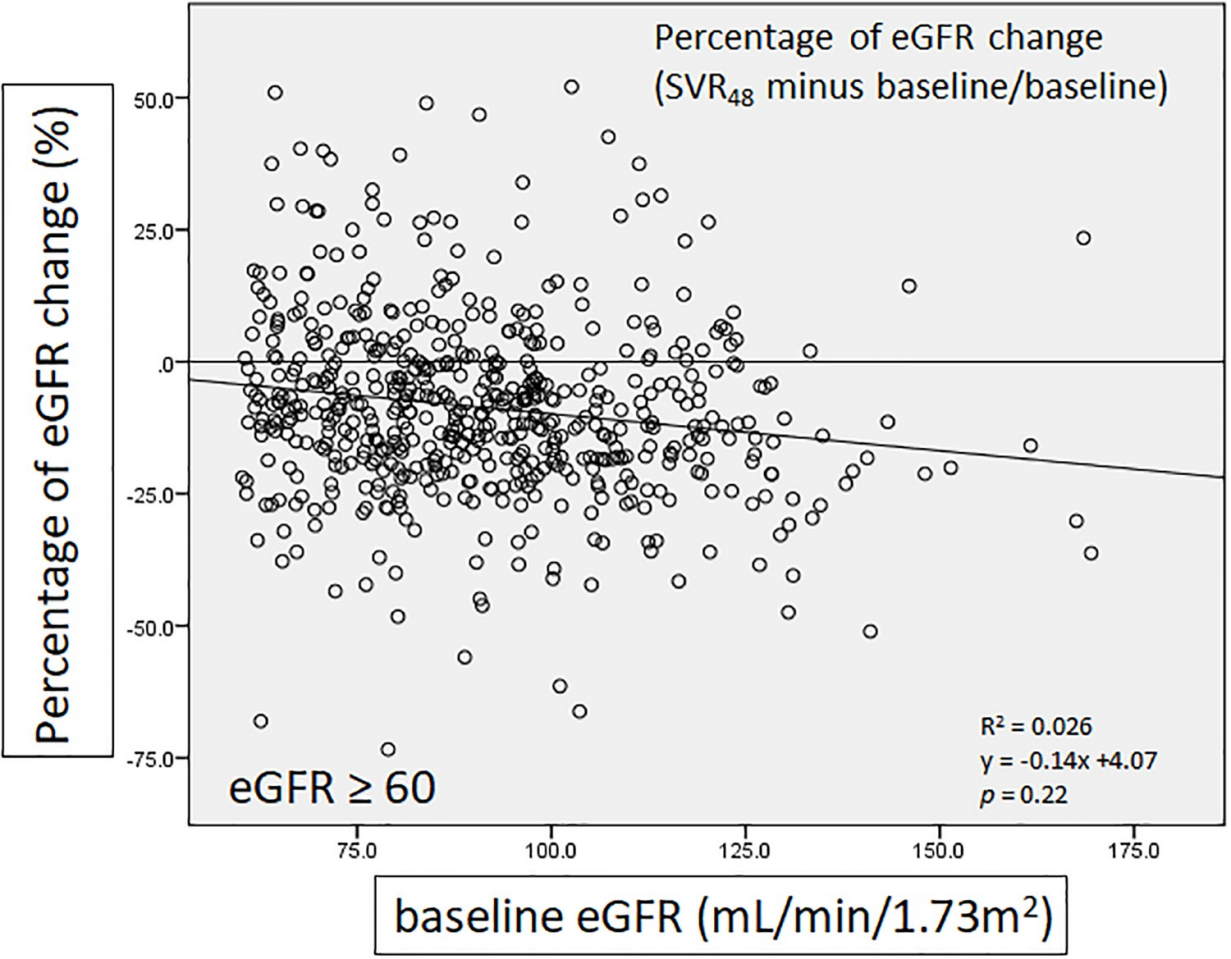
The correlation of percentage of eGFR change with baseline eGFR (baseline eGFR ≥ 60 mL/min/1.73m^2^).

**Fig 6:**
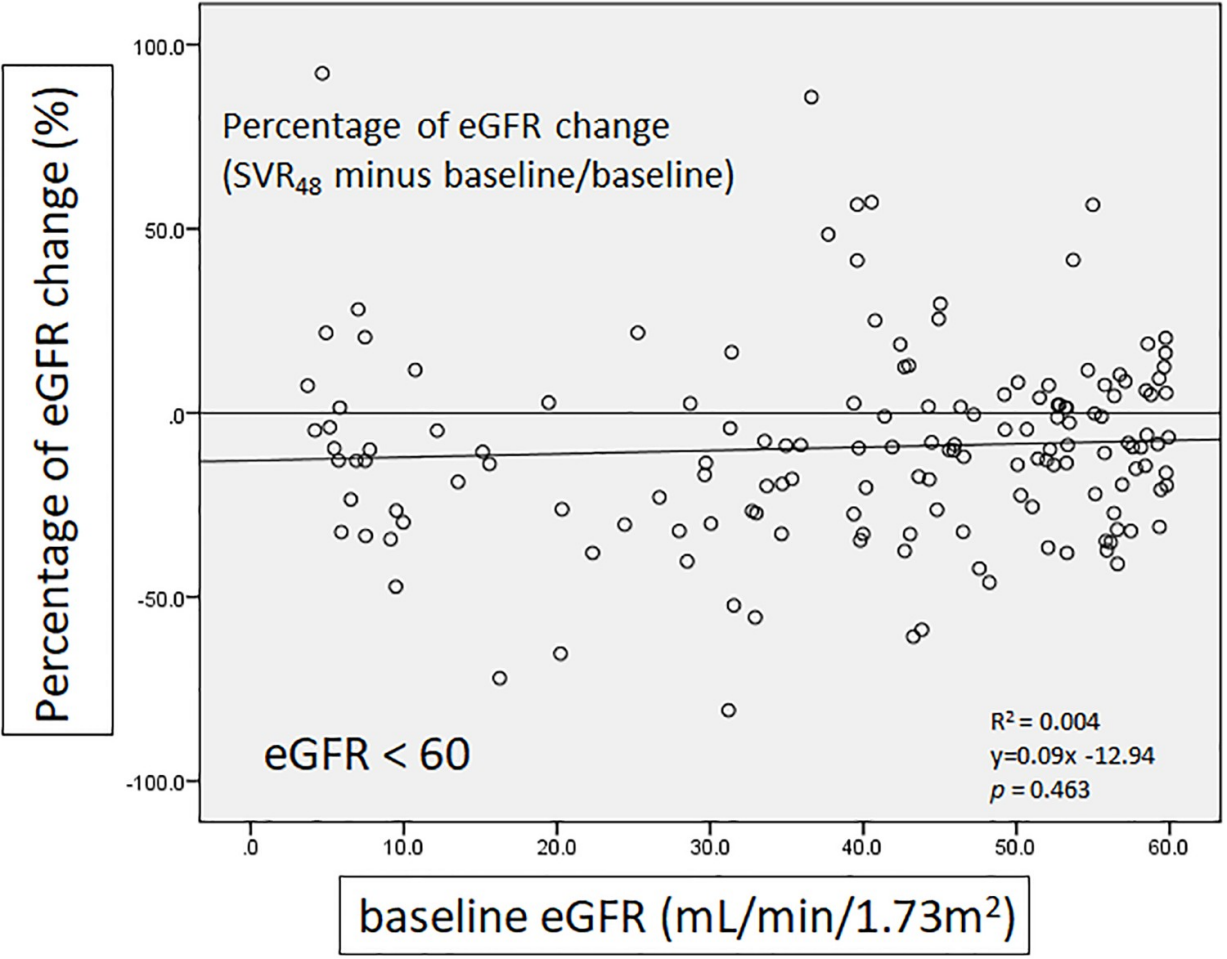
The correlation of percentage of eGFR change with baseline eGFR (baseline eGFR < 60 mL/min/1.73m^2^).

We defined percentage of eGFR change as SVR_48_ minus baseline then divided by baseline, in order to present the amplitude of change in eGFR. There was a significant negative correlation between percentage of eGFR change and baseline eGFR in the group of all patients (*p* = 0.01)(Fig 4). In patients with baseline eGFR ≥ 60 mL/min/1.73m^2^, negative correlation was also noted but there was no significant difference (*p* = 0.22)(Fig 5). In contrast, patients with baseline eGFR < 60 mL/min/1.73m^2^ showed positive association without significant difference (*p* = 0.463). (Fig 6)

### Univariate and multivariate analysis of predictive factors for deterioration of renal function

We further defined renal function deterioration as decrease of eGFR >25% from baseline to SVR_24_. Among all patients, 34 (34/831 = 4.09%) in ProD group, 12 (12/265 = 4.53%) in DCV/ASV group, 26 (26/222 = 11.7%) in SOF-based group and 14 (14/219 = 6.39%) in GRZ/EBR group suffered from deterioration of renal function from baseline to SVR_24_. Univariate analysis demonstrated that liver transplantation (OR = 4.468, 95% CI: 2.462–8.109, *p* < 0.001), hypertension (OR = 2.422, 95% CI: 1.563–3.752, *p* < 0.001), baseline eGFR < 60 mL/min/1.73m^2^ (OR = 2.785, 95% CI: 1.760–4.407, *p* < 0.001) and SOF-based DAA (OR = 1.955, 95% CI: 1.204–3.176, *p* = 0.007) were significant risk factors for deterioration of renal function from baseline to SVR_24_. Further multivariate analysis indicated that liver transplantation (OR = 5.378, 95% CI: 2.879–10.048, *p* < 0.001), hypertension (OR = 2.222, 95% CI: 1.379–3.582, *p* = 0.001) and baseline eGFR < 60 mL/min/1.73m^2^ (OR = 2.185, 95% CI: 1.333–2.580, *p* = 0.002) were independent predictive factors for deterioration of renal function from baseline to SVR_24_.(Table 2)

**Table 2:** Univariate and multivariate analysis of predictive factors for progression in renal function*.

		univariate		multivariate	
Variable	Comparison	OR (95%CI)	*p* value	OR (95%CI)	*p* value
**Age (years)**	> 65 vs. ≦ 65	1.218 (0.796–1.862)	0.364		
	> 60 vs. ≦ 60	0.881 (0.556–1.397)	0.59		
**Sex**	Male vs. Female	1.038 (0.679–1.586)	0.863		
**Liver cirrhosis**	Positive vs. Negative	0.81 (0.527–1.244)	0.335		
**HCC**	Positive vs. Negative	1.345 (0.780–2.322)	0.286		
**Diabetes mellitus**	Positive vs. Negative	1.134 (0.739–1.738)	0.566		
**Liver transplantation**	Positive vs. Negative	4.468 (2.462–8.109)	<0.001	5.378 (2.879–10.048)	<0.001
**Hypertension**	Positive vs. Negative	2.422 (1.563–3.752)	<0.001	2.222 (1.379–3.582)	0.001
**Baseline eGFR**	< 60 vs. ≥ 60	2.785 (1.760–4.407)	<0.001	2.185 (1.333–2.580)	0.002
**DAAs**	ProD vs. others	0.666 (0.434–1.022)	0.063		
	DCV/ASV vs. others	0.753 (0.409–1.385)	0.361		
	SOF-based vs. others	1.955 (1.204–3.176)	0.007		
	GRZ/EBR vs. others	1.209 (0.684–2.136)	0.514		

*Definition of progression in renal function: >25% decrease in eGFR from baseline to SVR_24_

Abbreviation: ProD, paritaprevir/ritonavir/ombitasvir/dasabuvir; DCV/ASV, daclatasvir/asunaprevir; SOF, sofosbuvir; GRZ/EBR, grazoprevir/elbasvir; AST, aspartate transaminase; ALT, alanine aminotransferase; HCC, hepatocellular carcinoma; eGFR, estimated glomerular filtration rate

**Fig 7:**
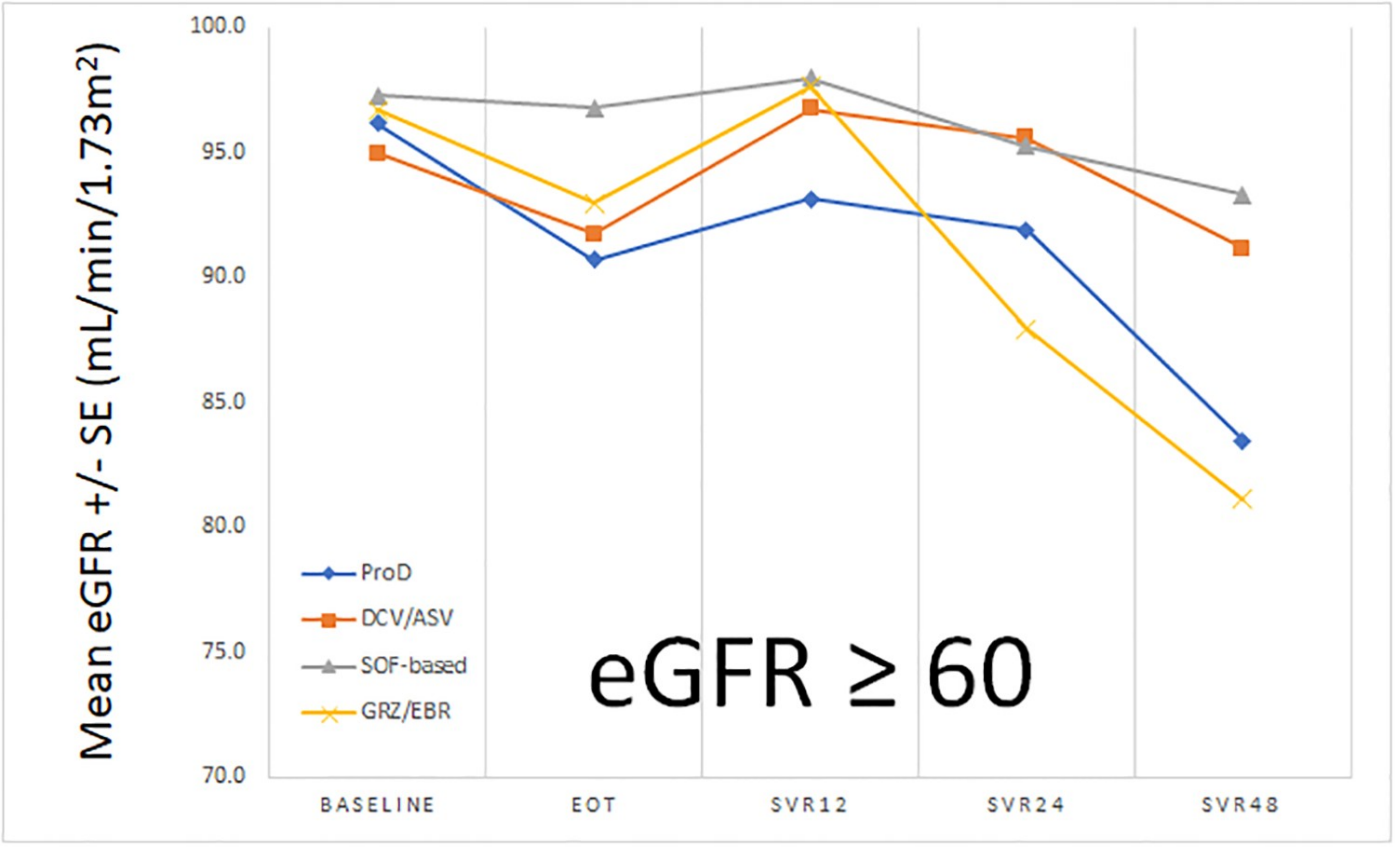
The trend of eGFR from baseline to SVR_48_ (exclude the patients with diabetes mellitus, hypertension, hepatocellular carcinoma and liver transplantation) (baseline eGFR ≥ 60 mL/min/1.73m^2^).

**Fig 8:**
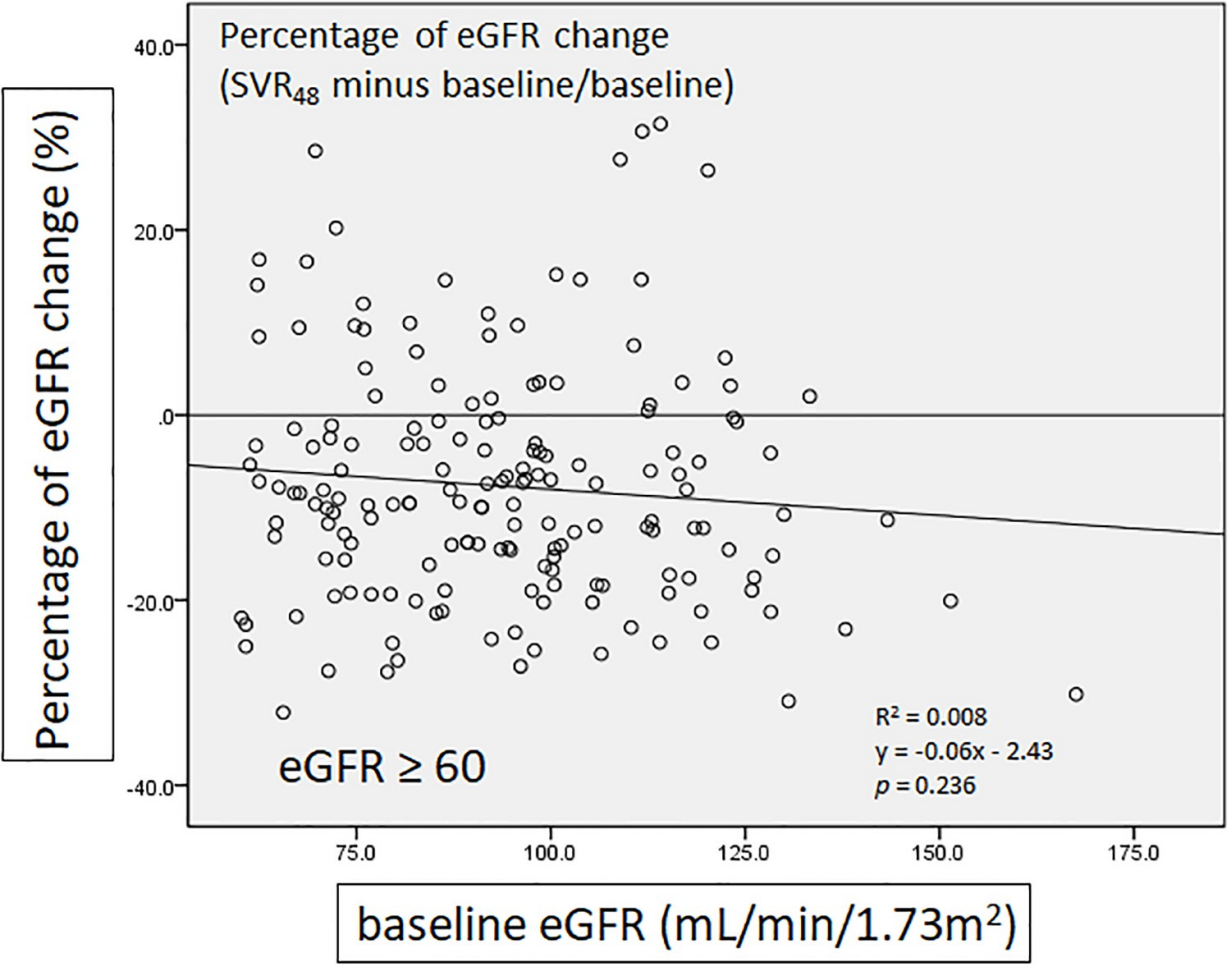
The correlation of percentage of eGFR change with baseline eGFR (exclude diabetes mellitus, hypertension, hepatocellular carcinoma and liver transplantation).

We further analyze persistent deterioration of renal function as decrease of eGFR >25% from baseline to SVR_48_. Among all patients, 45 (45/831 = 5.42%) in ProD group, 26 (26/265 = 9.81%) in DCV/ASV group, 28 (28/222 = 12.61%) in SOF-based group and 11 (11/219 = 5.02%) in GRZ/EBR group suffered from persistent deterioration of renal function from baseline to SVR_48_. Univariate analysis demonstrated that liver transplantation (OR = 2.430, 95% CI: 1.364–4.329, *p* = 0.003), hypertension (OR = 1.726, 95% CI: 1.151–2.587, *p* = 0.008), baseline eGFR < 60 mL/min/1.73m^2^ (OR = 2.540, 95% CI: 1.652–3.907, *p* < 0.001) and DCV/ASV use (OR = 1.606, 95% CI: 0.985–2.618, *p* = 0.058) were significant predictive factors for deterioration of renal function from baseline to SVR_48_. Further multivariate analysis indicated that liver transplantation (OR = 2.975, 95% CI: 1.628–5.434, *p* < 0.001), baseline eGFR < 60 mL/min/1.73m^2^ (OR = 2.624, 95% CI: 1.693–4.065, *p* < 0.001) and DCV/ASV use (OR = 1.826, 95% CI: 1.097–3.039, *p* = 0.021) were independent predictive factors for persistent deterioration of renal function from baseline to SVR_48_.(Table 3). Finally, we try to exclude patients with risk factors such as CKD patients at baseline, diabetes, hypertension, and liver transplantation for analysis again. We found that the trend of eGFR evolution showed a similar curve when all risk patients with diabetes mellitus, hypertension, hepatocellular carcinoma and liver transplantation were excluded (Fig 7). There is also negative correlation between percentage of eGFR change from baseline to the end of follow up after excluding diabetes mellitus, hypertension, hepatocellular carcinoma and liver transplantation. (Fig 8)

**Table 3:** Univariate and multivariate analysis of predictive factors for persistent progression in renal function*.

		univariate		multivariate	
Variable	Comparison	OR (95%CI)	*p* value	OR (95%CI)	*p* value
**Age (years)**	> 65 vs. ≦ 65	1.083 (0.728–1.612)	0.693		
	> 60 vs. ≦ 60	1.133 (0.714–1.797)	0.597		
**Sex**	Male vs. Female	0.954 (0.641–1.419)	0.816		
**Liver cirrhosis**	Positive vs. Negative	1.261 (0.832–1.909)	0.274		
**HCC**	Positive vs. Negative	1.564 (0.973–2.515)	0.065		
**Diabetes mellitus**	Positive vs. Negative	1.464 (0.982–2.184)	0.062		
**Liver transplantation**	Positive vs. Negative	2.430 (1.364–4.329)	0.003	2.975 (1.628–5.434)	<0.001
**Hypertension**	Positive vs. Negative	1.726 (1.151–2.587)	0.008		
**Baseline eGFR**	< 60 vs. ≥ 60	2.540 (1.652–3.907)	<0.001	2.624 (1.693–4.065)	<0.001
**DAAs**	ProD vs. others	0.721 (0.484–1.074)	0.108		
	DCV/ASV vs. others	1.606 (0.985–2.618)	0.058	1.826 (1.097–3.039)	0.021
	SOF-based vs. others	1.337 (0.835–2.140)	0.226		
	GRZ/EBR vs. others	0.695 (0.367–1.317)	0.265		

*Definition of progression in renal function: >25% decrease in eGFR from baseline to SVR_48_

Abbreviation: PrOD, paritaprevir/ritonavir/ombitasvir/dasabuvir; DCV/ASV, daclatasvir/asunaprevir; SOF, sofosbuvir; GRZ/EBR, grazoprevir/elbasvir; AST, aspartate transaminase; ALT, alanine aminotransferase; HCC, hepatocellular carcinoma; eGFR, estimated glomerular filtration rate

## Discussion

The present study demonstrated that there was a trend of decline of renal function at 1-year after DAA treatment. The trend was similar for all treatment regimens. There was a significant linear decrease of eGFR from baseline to SVR_48_ in patients with baseline eGFR ≥ 60 mL/min/1.73m^2^, but no significant difference was noted in those with baseline eGFR < 60 mL/min/1.73m^2^ even though there was still a trend of decline. In Taiwan, the reimbursement of DAA treatment for chronic HCV infection started in 2017. Few studies have investigated the issue of long-term renal safety after DAA treatment. Our study is the largest series with longest follow-up of renal function in the real world.

As we already knew, there is significant risk of experiencing CKD after HCV infection, with the lower eGFR as longer HCV exposed [9]. Theoretically, clearance of HCV should lead to improvement of renal function. However, the results in the present study go against the expectation. Our previous study has shown a slight decline of eGFR during the treatment of DAAs, followed by a slight rise 12 weeks after treatment (SVR_12_) [5]. But surprisingly, we found that eGFR declines again at SVR_24_ and SVR_48_. This is a novel finding that has not been reported. The decline of eGFR was evident even in patients without CKD at baseline and it was still there when all the risk patients including diabetes mellitus, hypertension, hepatocellular carcinoma and liver transplantation were excluded for analysis (Fig 8). So, the decline of eGFR during DAA treatment, as well as at time points of SVR_24_ and SVR_48_ might be a meaningful warning for all HCV patients after DAA treatment.

As we know, small fluctuations in eGFR are common and might not necessarily indicate deterioration of renal function. Some experts suggested that an assessment of >25% of decrease in eGFR was adopted to define renal function deterioration in clinical practice. Based on this definition, 626 (40.76%) patients suffered from decrease of eGFR from baseline to SVR_24_. But only 88 (5.73%) patients fitted the criteria of >25% of decrease in eGFR. Similarly, 357 (23.24%) patients developed decrease of eGFR from baseline to SVR_48_. Only 93 (6.05%) patients fitted the criteria. These results indicated that most deterioration of renal function might not be clinically significant. Of the 93 patients with >25% of decrease in eGFR from baseline to SVR_48_, none of them developed into end stage kidney disease and need dialysis until now. Therefore, longer follow-up study is needed to clarify this issue.

Except for patients with deterioration of renal function, some patients developed improvement of renal function at time of SVR_48_. Total 196 (12.63%) patients developed increase of eGFR from baseline to SVR_48_, which is less than the number of patients with decrease of eGFR. The multivariate analysis showed no significant predictive factors for renal function improvement. The possible explanation is too short follow-up period to express the power to predict the renal function improvement. For those patients with renal function improvement, the other possible reason is that the value of eGFR is affected mainly by serum creatinine value, age and sex. Creatinine is derived from creatine which is taken up by muscle [10]. As a result, increase or decrease in muscle mass may also influence the value of eGFR but not actual change of renal function. The study by Jeong-Ju Yoo *et al*. demonstrated that female sex, impaired liver function, and decreased muscle mass in males are independent risk factors of overestimation of renal function. Cystatin-C based eGFR is recommended to trace the renal function in these patients, especially in male patients with cirrhosis and sarcopenia [11]. In addition, Ryosuke Sugimoto et al also reported that HCV patients who undergone DAA treatment may induce the increase of muscle mass after treatment [12]. Increase of muscle mass may be also one of the reasons of decline of renal function in these patients, but we did not check muscle mass in our patients. In the future, further study of relationship between muscle mass and renal function in such patients is needed. In addition of creatinine and sex, age is also a possible factor affecting the renal function. But we did not find significant difference via multivariate analysis, the possible reason is that our follow-up period of 48 weeks is too short to see the change by age. So further study of longer follow-up period is needed to determine its relationship.

Among our regimens of DAA, sofosbuvir is the only DAA which is excreted by kidney. Many previous studies had noticed the renal safety of sofosbuvir-based DAA in patients with chronic kidney disease, but the results were controversial. Shin *et al* observed that four patients had worsening renal function after sofosbuvir-based DAA use, which included 2 out of 21 patients (9.5%) with CKD stage 3A and 2 out of 7 patients (28.6%) with CKD stage 3B [13]. Saxena *et al* also showed that sofosbuvir-based DAA treatment induced worsening of renal function in 29(2%) patients. Furthermore, patients with baseline eGFR ≤ 45 mL/min/1.73m^2^ was an independent risk factor for deterioration of renal function compared with patients with baseline eGFR > 45 mL/min/1.73m^2^ [14]. In contrast, Okubo *et al* reported that sofosbuvir-based therapy for genotype 1b chronic hepatitis C patients did not deteriorate serum creatinine levels, irrespective of baseline eGFR levels [15]. In addition to sofosbuvir-based DAA, other regimens of DAA were also discussed about renal safety in recent studies. Butt *et al*. reported a study of renal function from the ERCHIVES trials, where ProD group had higher proportion of eGFR decline [16]. Alvarez–Ossorio *et al*. demonstrated that only the subset of HIV-infected individuals showed significant decline in eGFR after ProD therapy for 12 weeks [17]. In the presented study, significant decline of renal function from baseline to SVR_48_ was noticed especially in SOF-based therapy, but it was not an independent risk factor of renal function deterioration. It is mandatory to suggest that clinical physicians should closely monitor the renal function in SOF-based treated patients.

In the present study, multivariate analysis for the deterioration (decrease of eGFR > 25% from baseline to SVR_24_) of renal function revealed that liver transplantation, hypertension and baseline eGFR < 60 mL/min/1.73m^2^ are predictive factors. It is reasonable that patients underwent liver transplantation have received different kinds of immunosuppressive agents, which might affect renal function. Multivariate analysis for persistent deterioration (a decrease of eGFR >25% from baseline to SVR_48_) of renal function showed liver transplantation, baseline eGFR < 60 mL/min/1.73m^2^ and DCV/ASV use are risk factors. The possible explanation for DCV/ASV risk was unclear. We supposed it is related to longer treatment duration than other regimens: the mean treatment duration of DCV/ASV was 24 weeks; in contrast, the mean treatment duration of other DAAs were 8 to 12 weeks. In other recent studies, Sise M. E. *et al* also reported that diabetes mellitus was a predictive factor of deterioration of renal function in the cohort with stage 3 chronic kidney disease [18], which might be a reasonable change in diabetic patients. Taken together, regardless of DAAs regimens, patients with multiple systemic diseases have higher risk for renal function deterioration after DAA treatment in this study.

It is interesting that baseline eGFR <60 mL/min/1.73m^2^ is an independent risk factor for renal function deterioration. In fact, patients with baseline eGFR <60 mL/min/1.73m^2^ had less linear decrease in eGFR when compared with those of baseline eGFR ≥60 mL/min/1.73m^2^. So, the possible reason is that the proportion of renal function deterioration in subgroup of baseline eGFR <60 mL/min/1.73m^2^ is higher than that of the subgroup of baseline eGFR ≥60 mL/min/1.73m^2^ (15% v.s.5%). The results indicate again that the decline of eGFR less than 25% might not be clinically relevant. Longer period of observation is needed to clarify this issue.

There are limitations in our study. First, it is a retrospective study that there is missing data in some of the patients during follow up. Second, there is a lack of control group of patients who are not treated with DAA, as well as patients treated with interferon-based therapies. Further prospective case controlled study is warranted to clarify this issue.

## Conclusions

In our study, there is a decline of eGFR at 1-year after DAAs treatment. Liver transplantation and baseline eGFR < 60 mL/min/1.73m^2^ are independent risk factors of the deterioration of renal function. We suggested that it is important for patients with risk factors to receive regular renal function test after DAA treatment.

## Supporting information

S1 Data (XLSX)

S2 Data (PDF)
