# Supplementary material for: Serial changes of renal function after directly acting antivirals treatment for chronic hepatitis C: A 1-year follow-up study after treatment
Source: PLoS One. 2020 Apr 14;15(4):e0231102. doi: 10.1371/journal.pone.0231102 (PMC7156075; doi:10.1371/journal.pone.0231102)
Supplement: S2 Data — (PDF) [file pone.0231102.s002.pdf]

長庚醫療財團法人人體試驗倫理委員會
臨床試驗同意證明書

地　　址：105台北市敦化北路199號
傳　　真：03-3494549
聯絡人及電話：張正怡(03)3196200#3713
電子郵件信箱：ccyi@cgmh.org.tw

試驗名稱： 直接抗病毒藥物治療C型肝炎病患對腎功能的影響：真實世界的經驗
本院案號： 201900673B0
試驗期間： 2019年5月17日~2019年6月16日

本次核准執行期間：2019年5月17日~2019年6月16日
主持人： 胃腸肝膽科系 蔡明釗 學術組助理教授級主治醫師
共同主持人： 林俊彥,胡琮輝,簡榮南,張國欽,洪肇宏
執行機構： 基隆長庚醫院,林口長庚醫院,嘉義長庚醫院,高雄長庚醫院
核准之計畫書版本： 2019/05/09 Version2
核准之同意書版本： 本案經本會同意免除受試者同意書

本案『使用臨床常規治療或診斷之病歷，含個案報告之研究。但不含人類後天性免疫不全病毒（HIV）陽性患者之病歷。』，經本會判斷為簡易審查

通過日期：2019年5月17日

※本研究將於2019年6月16日到期，請於試驗到期後三個月內繳交結案報告至本會審查，若需要展延試驗期間者，請於到期前兩個月提出變更案，並繳交期中報告。

本委員會組織與運作皆遵守GCP規定

長庚醫療財團法人
人體試驗倫理委員會謝燦堂主席

長庚醫療財團法人研究計畫專用(2)

2019　年　05　月　17　日

Chang Gung Medical Foundation

Institutional Review Board

199, TUNG HWA NORTH ROAD,
TAIPEI, TAIWAN, 10507
REPUBLIC OF CHINA
Tel: (03) 3196200
Fax: (03) 3494549

Date 2019/05/17

Protocol Title: Revolution of Renal function under directly acting antivirals treatment for chronic hepatitis C: A real world experience

IRB No.: 201900673B0

Principal Investigator(s): Tsai, Ming-Chao

Co-Investigator(s): LIN-CHUN-YEN, HU, TSUNG-HUI, CHIEN, RONG-NAN, Chang, Kuo-Chin, HUNG, CHAO-HUNG

Executing Institution: Keelung, Linkou, Chiayi, Kaohsiung

Duration of Approval: From 2019/05/17 TO 2019/06/16

Approved Protocol: 2019/05/09 Version2

The IRB reviewed and determined that it is expedited review according to Case research or cases treated or diagnosed by clinical routines. However, this does not include HIV positive cases.

Date of Approval: 2019/05/17

Frequency of Continuing Report: Once a year

※The research will expire on 2019/06/16. Please submit the final report to the IRB for review within three months after the expiration of the trial research. For extension of the trial period, please submit the amendment and a continuing report two months before the expiration.

The IRB is organized and operates in accordance with Good Clinical Practice and the applicable laws and regulations.

Tsang-Tang Hsieh,MD
Chairman
Institutional Review Board
Chang Gung Medical Foundation

【主持人須知】

一、 實施人體研究計畫前，應擬定研究計畫，經人體試驗倫理委員會審查通過，始得為之。另醫療法所稱之人體試驗案及應用人體生物資料庫檢體進行之案件，尚需經衛生福利部核准，方可進行。人體試驗倫理委員會或主管機關命令中止/終止試驗案件時，不得繼續執行。

二、 廠商贊助試驗於執行前，應協助試驗執行醫院完成臨床試驗合約書簽署，並提供本會影本乙份。合約書簽署流程及注意事項應依本院之「廠商贊助計畫管理作業準則」辦理。展延試驗期間者，經人體試驗倫理委員會同意後，應進行合約變更。

三、 試驗進行前，主持人應確實核對試驗計畫書、受試者同意書等之正確版本，以及人體試驗倫理委員會與衛生福利部核准之試驗進行期間；醫療法所稱之人體試驗案，應於本院人體試驗倫理委員會與衛生福利部皆已核准，方可進行；並以人體試驗倫理委員會核准之同意臨床試驗證明迄日為試驗截止日。

四、 應完全熟悉試驗藥品/醫療材料、醫療技術在試驗計畫書、最新主持人手冊及其他由試驗委託者提供的相關資訊中描述的使用方法。

五、 應明瞭並遵守「醫療法」、「人體試驗管理辦法」、「人體研究法」、「人體生物資料庫管理條例」、「藥品優良臨床試驗準則」、「醫療器材優良臨床試驗準則」等相關法規，以及本院「人體試驗作業管理辦法」、「人體生物資料庫管理辦法」、「研究材料外送作業準則」等之規定，善盡保護受試者之責任，並配合主管機關、人體試驗倫理委員會及執行機構的查核。

六、 應確保所有協助臨床試驗的相關人員，對試驗計畫書及試驗藥品/醫療材料、醫療技術有充分的了解，以及他們在臨床試驗中相關的責任和工作，並定期召開會議討論。

七、 試驗主持人應負責所有臨床試驗相關的醫療決定。

八、 在受試者參加試驗與後續追蹤期間，試驗主持人應對受試者任何與試驗相關的不良反應，提供充分的醫療照護，包括重要實驗室檢查值等。當試驗主持人察覺試驗期間受試者有疾病需要醫療照護時，必須告知受試者。

九、 主持人須遵照「嚴重藥物不良反應通報辦法」及「藥品優良臨床試驗準則」、「人體試驗管理辦法」之規定，於時效內向相關單位完成通報。發生非預期且相關之嚴重藥品不良反應，試驗主持人應立即通知人體試驗倫理委員會，如為衛生福利部列管案件，並需確認試驗委託者已通報主管機關；如為主持人自行發起之案件，主持人需通報主管機關。但若試驗計畫書或其他文件明確排除者，不在此限。如為已上市藥品試驗案發生藥品不良反應（ADR），應依規定至院內網頁之安全通報作業系統完成通報。

十、 試驗主持人應依本院「人體試驗作業管理辦法」規定繳交期中報告及結案報告，未繳者不得再申請新案，案件審議如有必要時須配合於會議列席報告。

十一、 受試者同意書使用原則須遵照「藥品優良臨床試驗準則」之規定，若具有重要性之新資訊可能影響受試者之同意時，應修訂受試者同意書及提供受試者之任何其他書面資料，並應立即告知受試者、法定代理人或有同意權之人，並重新取得書面同意。修訂後之受試者同意書及提供受試者之任何其他書面資料，應先得到人體試驗倫理委員會之核准；經主管機關核准進行之臨床試驗，並應得到主管機關之核准。

十二、 經人體試驗倫理委員會核准之計畫內容，凡於核准期間內任一變更(包含試驗計劃書、主持人手冊、受試者同意書、問卷、量表、執行院區、計畫主持人、試驗委託者等)，應於執行前送變更案審查，於接獲書面核准函後，始可為之。另計劃主持人因故自行暫停、終止或結案時，應以書面通知本會。

十三、 試驗研究團隊執行試驗時發生未遵照審查通過之計畫書、主管機關所訂立之法令規章或本會規定之情事，應遵照本院「人體試驗作業管理辦法」之規定，於時效內完成通報。

十四、 人體試驗倫理委員會同意之試驗期間到期，但需要展延試驗期間者，應於有效期限到期前2個月提出申請。

十五、 醫療法所稱人體試驗範圍之案件，侵入性檢查或治療，以及使用上市藥品之案件需至『HIS系統-研究計畫-研究計畫申請-臨床試驗執行管控作業』登錄受試者資料。

十六、 試驗主持人於門診(住院)時發現受試者發生與試驗相關的不良反應，需轉診時，試驗主持人應主動向受試者及家屬解釋，並協助轉診(會診)，且於病歷(會診單)上詳細記載本試驗目的、副作用、目前受試者病情及處置，並口頭告知轉診主治醫師。

十七、 若為多國多中心之案件，計畫主持人須確認第一個Kit之正確性。

十八、 應遵照「人體試驗管理辦法」、「醫療機構及醫事人員發布醫學新知或研究報告倫理守則」及本院「研究成果發表於報章媒體作業準則」規定，醫療法所稱人體試驗之研究成果須經衛生福利部審核通過後，試驗主持人始得發表。

十九、 因試驗計畫主持人過失造成醫院或他人受損害時，須由計畫主持人負法律責任。

二十、 免除受試者同意後仍會適時提供受試者試驗相關訊息。

二十一、 當受試者在納入研究後成為受刑人，主持人得知後應以非預期問題通報本會及試驗委託者。

二十二、 其餘未盡事宜，請參照本院「人體試驗作業管理辦法」辦理。

二十三、 試驗團隊於試驗執行時需遵守事項：臨床試驗執行需於病歷記載、於病房收案需向護理人員進行教育訓練及需填寫試驗團隊工作職責分配表、每位計畫主持人執行廠商贊助計畫件數不得超過10件。

CHANG GUNG MEDICAL FOUNDATION
199 TUNG HWA NORTH ROAD
TAIPEI, TAIWAN, 10507
REPUBLIC OF CHINA
TEL(03)3196200
FAX(03)3494549

Institutional Review Board A

2017 July - 2019 June

MEMBER	GENDER	PRIMARY SPECIALTY	AFFILIATION OF INSTITUTION
主席 : 謝燦堂<br>HSIEH, TSANG-TANG	Male<br>男性	Obstetrics/Gynecology<br>婦產科專科	CGMF (Taipei)<br>台北長庚醫院
副主席 : 陳順勝<br>SHUN--SHENG-CHEN	Male<br>男性	Neurology<br>神經內科專科	CGMF (Kaohsiung)<br>高雄長庚醫院
醫療執秘 : 蘇建安<br>SU, JIAN-AN	Male<br>男性	Psychiatry<br>精神科專科	CGMF (Jiayi)<br>嘉義長庚醫院
鄭欽明<br>CHENG, CHIN-MING	Male<br>男性	Business Management<br>企業管理	Children's Hearing Foundation<br>財團法人雅文兒童聽語文教基金會
黃英霓<br>Ying Ni Huang	Female<br>女性	Law<br>法律	None<br>無
傅懋洋<br>FU, MAO-YOUNG	Male<br>男性	Cardiology<br>心臟內科專科	CGMF (Kaohsiung)<br>高雄長庚醫院
郭亮鍪<br>KUO, LIANG-MOU	Male<br>男性	General Surgery<br>一般外科專科	CGMF (Jiayi)<br>嘉義長庚醫院
黃怡婷<br>Yi-Ting Hwang	Female<br>女性	Statistics<br>統計	National Taipei University<br>國立臺北大學
張毓翰<br>CHANG,YU-HAN	Male<br>男性	Orthopaedic<br>骨科	CGMF (Lin-Kou)<br>林口長庚醫院
林欣柔<br>SHIN-ROU LIN	Female<br>女性	Law<br>法律	Chang Gung University<br>長庚大學
沈一嫻<br>I-Shyan Sheen	Female<br>女性	HepatoGastroenterology<br>胃腸肝膽科專科	CGMF (Lin-Kou)<br>林口長庚醫院
廖繼洲<br>Chi-Chou Liao	Male<br>男性	Pharmacy<br>藥事	None<br>無
王正旭<br>WANG,CHENG-HSU	Male<br>男性	Hematology/Oncology<br>腫瘤科專科	CGMF (Kee-Lung)<br>基隆長庚醫院
曾旭民<br>Hsu-Min Tseng	Male<br>男性	Psychology<br>心理學	Chang Gung University<br>長庚大學
游靜宜<br>YU,CHING-YI	Female<br>女性	Social Work<br>社工	CGMF (Lin-Kou)<br>林口長庚醫院

Chairman:

Tsang-Tang Hsieh,MD
